# Effects of Androgen Receptor Inhibition on Kanamycin-Induced Hearing Loss in Rats

**DOI:** 10.3390/ijms22105307

**Published:** 2021-05-18

**Authors:** Kyung-Ju Chun, Chang-Ho Lee, Kyung-Woon Kim, So-Min Lee, So-Young Kim

**Affiliations:** Department of Otorhinolaryngology-Head & Neck Surgery, CHA University College of Medicine, Seongnam 13496, Korea; a206015@chamc.co.kr (K.-J.C.); hearwell@gmail.com (C.-H.L.); wgaltz@naver.com (K.-W.K.); lws6812@naver.com (S.-M.L.)

**Keywords:** receptors, androgen, aminoglycosides, hearing loss, low-density lipoprotein receptor-related protein-2, metallothionein

## Abstract

Megalin has been proposed as an endocytic receptor for aminoglycosides as well as estrogen and androgen. We aimed to investigate the otoprotective effects of antiandrogens (flutamide, FM) on kanamycin (KM)-induced hearing loss in rats. Rats were divided into four groups. The KM group was administered KM (20 mg/kg/day) for 5 days, while the FM group received FM (15 mg/kg/day) for 10 days. In the KM + FM group, KM and FM (15 mg/kg/day) were simultaneously injected for 5 days and then FM was injected for 5 days. Auditory brainstem responses were measured. Western blotting and/or quantitative reverse transcriptase-polymerase chain reaction were performed for megalin, cytochrome P450 1A1 (Cyp1a1), Cyp1b1, metallothionein 1A (MT1A), MT2A, tumor necrosis factor (TNF)-α, caspase 3, and cleaved caspase 3. The FM + KM group showed attenuated auditory thresholds when compared with the KM group at 4, 8, 16, and 32 kHz (all *p* < 0.05). The KM + FM group showed lower megalin and Cyp1b1 levels than the KM group (all *p* < 0.05). The KM + FM group revealed lower MT1A, TNFα, and caspase 3 protein levels, compared with those in the KM group (all *p* < 0.05). Androgen receptor inhibition protects against cochlear injuries in KM-induced hearing loss rats by attenuating megalin expression, revealing anti-inflammatory and anti-apoptotic effects.

## 1. Introduction

Although aminoglycosides have beneficial effects in antibacterial therapy, they can induce irreversible sensorineural hearing loss. Aminoglycoside-induced hearing loss involves oxidative stress and inflammatory responses [1]. Aminoglycosides can reportedly enter both sensory hair cells and supporting cells via mechanotransducer channels and accumulated intracellular aminoglycosides complex with iron, inducing the synthesis of reactive oxygen species (ROS) [2,3]. ROS formation promotes several pro-inflammatory cascades involving tumor necrosis factor α (TNFα) and caspase 3 activation [1]. Several reports have indicated that otoprotective drugs possess antioxidative effects. However, there is no available clinical treatment for aminoglycoside ototoxicity [4]. Additionally, drugs that inhibit the transportation of ototoxic drugs have been proposed for treating aminoglycoside ototoxicity [4,5].

Megalin has been suggested as an endocytic aminoglycoside receptor [6]. Megalin is a low-density lipoprotein receptor transmembrane protein [6]. It functions as an endocytic receptor for several lipophilic ligands, including steroid hormones such as estrogen and androgen [7]. On interacting with diverse lipophilic metabolites, megalin regulates hormone metabolism and mediates intracellular signal transduction [8]. In vitro and in vivo studies have revealed that megalin mediates aminoglycoside-induced nephrotoxicity, and inhibition of megalin-mediated aminoglycoside endocytosis can reduce nephrotoxicity [9]. In the cochlea, megalin is expressed in multiple regions, including marginal cells of the stria vascularis, epithelial cells of the spiral prominence, and Reissner’s membrane [10]. Thus, it can be presumed that megalin might be involved in endocytosis of aminoglycoside in the cochlea in that it could mediate the aminoglycoside-induced ototoxicity. However, there has been a lack of study which explores the changes of megalin expression and the effects of megalin inhibition in an ototoxicity model.

A rat study has reported that megalin inhibition by androgen blockade affords protective effects against aminoglycoside-induced nephrotoxicity [11]. The study revealed the presence of several response elements to androgen receptors in promoter regions of megalin, implying the transcriptional regulation of megalin by androgen receptors [11]. Since a few previous studies suggested the sex differences in aminoglycoside-induced ototoxicity as well as megalin also exists in the cochlea, the suppression of megalin by androgen antagonist could have otoprotective effects in an aminoglycoside-induced ototoxicity model [10,12,13]. This study hypothesized that megalin inhibition by an androgen blocker such as flutamide (FM) might prevent aminoglycoside-induced ototoxicity. To test this hypothesis, aminoglycoside-induced hearing loss rats were co-treated with FM. These FM and aminoglycoside co-treated rats were compared with aminoglycoside-induced hearing loss rats. The auditory hearing thresholds, the pathology of the cochlea, and changes in gene expression levels related to oxidative stress and inflammation were evaluated to determine the effects of FM treatment on aminoglycoside-induced ototoxicity. To the best of our knowledge, limited data is available on the impact of androgen blocking on ototoxicity.

## 2. Results

Pre- and post-treatment auditory thresholds did not differ between the control and FM groups (Table 1). In the KM group, auditory thresholds were increased post-treatment at all examined frequencies of 4, 8, 16, and 32 kHz (all *p* < 0.05). Post-treatment, auditory thresholds were lower in the KM + FM group than in the KM group (all *p* < 0.05).

**Figure 1:**
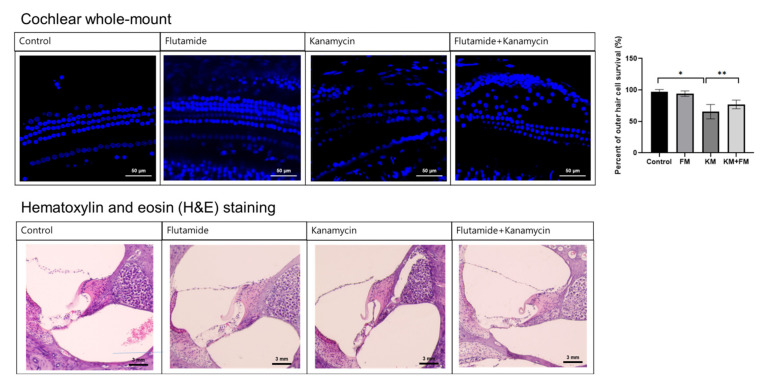
The cochlear whole-mount and hematoxylin and eosin (H&E) staining of the cochlea. The FM + KM group showed smaller changes in the loss of outer hair cells and spiral ganglion cells than the KM group (* *p* < 0.05 in unpaired *t*-test between control and KM groups, ** *p* < 0.05 in unpaired *t*-test between KM and KM + FM groups).

In cochlear whole-mount examinations, some loss of cochlear outer hair cells was observed, along with disorientation of cochlear outer hair cell arrangements in the KM group (Figure 1). The percentage of intact outer hair cells was less in the KM group compared to the control group (*p* < 0.001 in ANOVA and *p* = 0.002 in unpaired *t*-test). Although the FM + KM group also demonstrated the loss and disorientation of outer hair cells, they showed smaller changes in the loss of outer hair cells than the KM group (*p* = 0.004 in unpaired *t*-test). Hematoxylin and eosin (H&E) staining revealed spares of spiral ganglion cells in addition to outer hair cell injuries in the KM group.

**Figure 2:**
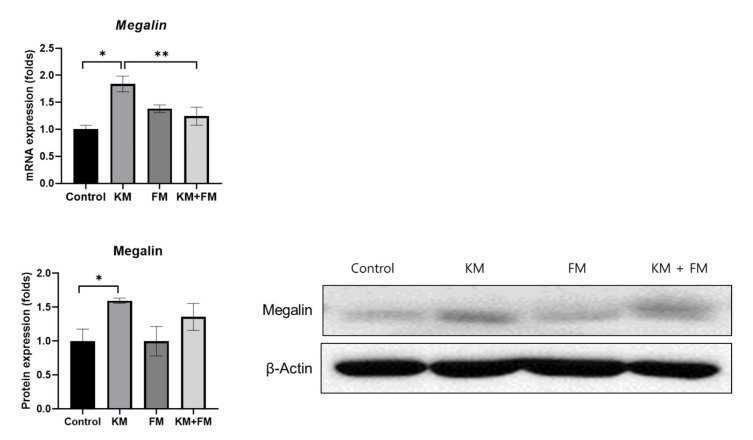
The mRNA and protein expression levels of megalin. In the KM + FM group, the mRNA level of megalin was lower than that observed in the KM group (* *p* < 0.05 in unpaired *t*-test between control and KM groups, ** *p* < 0.05 in unpaired *t*-test between KM and KM + FM groups).

Both mRNA and protein expression levels of megalin were higher in the KM group than in the control group (*p* = 0.001 and 0.049 in ANOVA among control, FM, KM, and KM + FM groups) (Figure 2). In the KM group, the mRNA level of megalin was 1.84-fold higher than that of the control group (standard deviation (SD) = 0.15, *p* = 0.001 in unpaired *t*-test), and the protein level was 1.60-fold higher than that in the control group (SD = 0.04, *p* = 0.011 in unpaired *t*-test). In the KM + FM group, the mRNA level of megalin was lower than that observed in the KM group (1.24-fold, SD = 0.17, *p* = 0.027 in unpaired *t*-test). The protein level of megalin was lower in the KM + FM group than that in the KM group; however, this difference was not significant.

**Figure 3:**
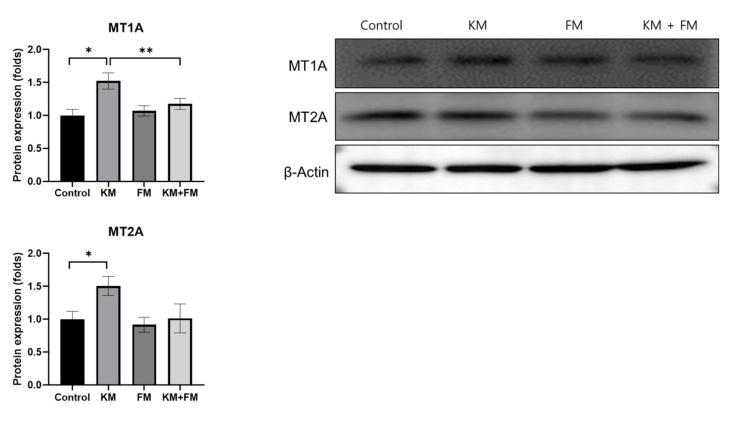
The protein expression levels of metallothionein 1A (MT1A) and MT2A. The KM + FM group showed lower levels of MT1A than the KM group (* *p* < 0.05 in unpaired *t*-test between control and KM groups, ** *p* < 0.05 in unpaired *t*-test between KM and KM + FM groups).

The KM group showed 1.52-fold (SD = 0.12, *p* = 0.01 in unpaired *t*-test) and 1.51-fold (SD = 0.14, *p* = 0.028 in unpaired *t*-test) higher protein levels of MT1A and MT2A when compared with those of the control groups (Figure 3). The KM + FM group showed lower levels of MT1A than the KM group (1.18-fold, SD = 0.08, *p* = 0.048 in unpaired *t*-test).

**Figure 4:**
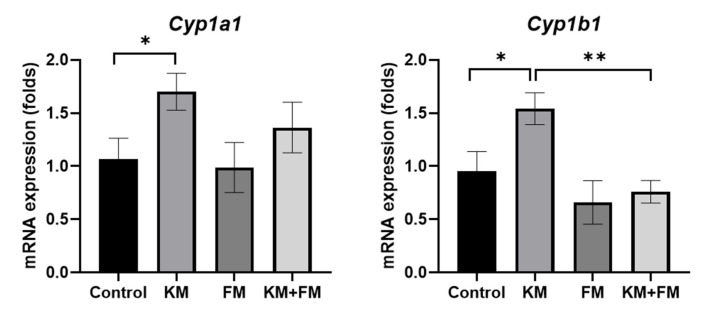
The mRNA expression of cytochrome P450 1A1 (Cyp1a1) and Cyp1b1. mRNA levels of Cyp1b1 were lower in the KM + FM group than in the KM group (* *p* < 0.05 in unpaired *t*-test between control and KM groups, ** *p* < 0.05 in unpaired *t*-test between KM and KM + FM groups).

The mRNA expression of *Cyp1a1* and *Cyp1b1* was 1.70-fold (SD = 0.17, *p* = 0.049 in ANOVA and 0.036 in unpaired *t*-test) and 1.54-fold (SD = 0.15, *p* = 0.006 in ANOVA and 0.034 in unpaired *t*-test) higher in the KM group than in the control group, respectively (Figure 4). mRNA levels of *Cyp1b1* were lower in the KM + FM group than in the KM group (0.76-fold, SD = 0.11, *p* = 0.002 in unpaired *t*-test).

**Figure 5:**
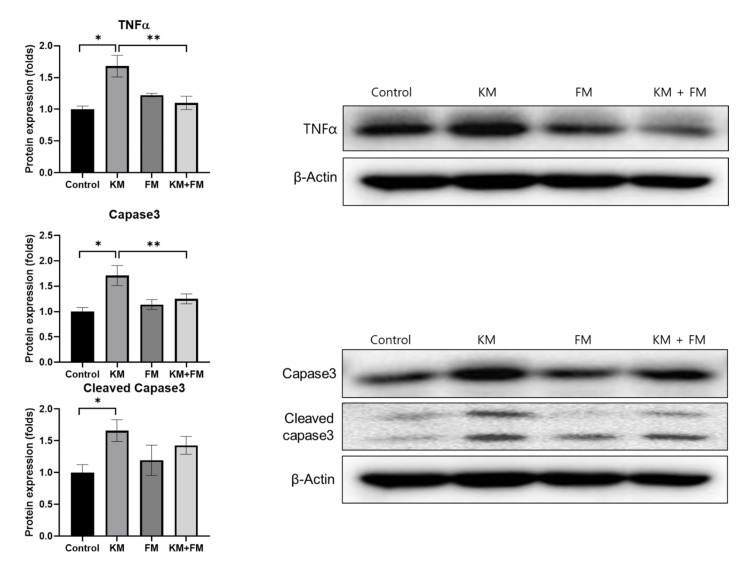
The protein expression levels of tumor necrosis factor (TNF)α, caspase 3, and cleaved caspase 3. The KM + FM group showed lower levels of TNFα and caspase 3 than the KM group (* *p* < 0.05 in unpaired *t*-test between control and KM groups, ** *p* < 0.05 in unpaired *t*-test between KM and KM + FM groups).

The protein expression of TNFα was higher in the KM group than in the control group (1.68-fold, SD = 0.17, *p* = 0.001 in ANOVA and 0.003 in unpaired *t*-test) (Figure 5). The KM + FM group demonstrated lower protein levels of TNFα than the KM group (1.10-fold, SD = 0.11, *p* = 0.014 in unpaired *t*-test). For caspase 3 and cleaved caspase 3, protein levels were 1.71-fold (SD = 0.20, *p* = 0.005 in ANOVA and 0.008 in unpaired *t*-test) and 1.66-fold (SD = 0.17, *p* = 0.049 in ANOVA and 0.021 in unpaired *t*-test) higher in the KM group than in the control group. In the KM + FM group, the protein level of caspase 3 was lower than that in the KM group (1.25-fold, SD = 0.10, *p* = 0.05 in unpaired *t*-test).

## 3. Discussion

In the present study, KM-induced hearing loss rats revealed increased expression of genes related to oxidative stress, inflammation, and apoptosis, including *Cyp1a1, Cyp1b1*, TNFα, caspase 3, and cleaved caspase 3. Furthermore, the expression levels of MT1A and MT2A and transmembrane megalin receptors were elevated in rats presenting KM-induced hearing loss. Administration of an androgen receptor antagonist, a known megalin ligand, attenuated KM-induced auditory threshold shifts, as well as expression levels of megalin, MT1A, *Cyp1b1*, TNFα, and caspase 3. The present results may improve previous findings by expanding the protective effects of FM from aminoglycoside-induced nephrotoxicity to aminoglycoside-induced ototoxicity. To the best of our knowledge, the application of androgen antagonists and the exploration of their protective mechanisms in hearing loss have not been previously reported. A few previous studies investigated the effects of testosterone on auditory functions [12,13]. The effects of testosterone in the immune-mediated sensorineural hearing loss rat models were suggested [12]. However, low level of testosterone did not change the auditory brainstem response and otoacoustic emissions in the UV-filter octyl methoxycinnamate-exposed rats [13]. Thus, it can be presumed that androgen or testosterone has a role in auditory function, but its effect could be different according to the pathophysiologic mechanisms of hearing loss, such as aminoglycoside-induced hearing loss or autoimmune-mediated hearing loss. Further studies are warranted to elucidate the effects of antiandrogen in specific types of hearing loss.

FM, an antiandrogen, decreased the expression level of megalin, which was elevated after KM administration. As megalin has been suggested as an endocytic aminoglycoside transporter, KM administration may induce megalin upregulation [9]. The androgen receptor reportedly regulates megalin expression [11]. Thus, androgen receptor blockade with FM could downregulate megalin expression in the KM + FM group. The elevated expression of megalin in the KM group can induce cochlear dysfunction through a few plausible molecular pathways, although the exact pathophysiology remains poorly defined. Megalin may impact hearing functions by regulating endolymphatic homeostasis via its multiligand endocytic functions [14]. Moreover, homeostasis of endolymphatic flow is essential for maintaining inner ear function. As megalin is expressed in multiple inner ear regions, it can influence endolymph homeostasis in the inner ear. Megalin is located in widespread regions of the inner ear, including the apical surface of the strial marginal cells, the epithelial cells of Reissner’s membrane facing the cochlear duct, spiral prominence, and endolymphatic sac, and transitional and dark cells of the utricle and semicircular canals [7,10,14]. Furthermore, changes in megalin expression could influence the inner ear function by modulating the otoprotective effects of estrogen. A study utilizing a megalin knockout transgenic mouse has revealed that megalin mediates the effects of estrogen in the cochlea by regulating estrogen endocytosis [7]. The otoprotective effects of estrogen have been reported and are found to be transduced through multiple estrogen receptors [15]. Additionally, estrogen replacement therapy reportedly prevented noise-induced hearing loss in ovariectomized rats [16].

In the present study, expression levels of MT1A and MT2A were increased in rats with KM-induced hearing loss and were normalized in KM + FM-treated rats. The increased expression of megalin induces ototoxicity via interaction with metallothioneins (MTs). MTs are reported ligands of megalin [17]. MTs are cysteine-rich zinc-binding proteins that act as antioxidants by suppressing the oxidative stress response of mitochondria [18]. The MT1A expression level was altered under hypoxic conditions in the organ of Corti, modiolus, and stria vascularis, as well as in spiral ligaments in a rat tissue culture study [19]. Under hypoxic culture conditions, *Mt1a* expression levels were increased in the organ of Corti [19]. In addition to antioxidant effects, MTs are considered reactive proteins that possess neuroprotective and regenerative effects [20]. In a mouse model of cuprizone-induced neurotoxicity, the expression levels of MT1/MT2 and megalin were increased in certain brain regions [21]. Thus, the increased expression of megalin and MT1A and MT2A ligands can induce oxidative and inflammatory responses in KM rats; in the present study, this was reversed in KM + FM rats by blocking the signaling cascades of the androgen receptor, megalin, and MT1A.

The attenuation of inflammation and oxidative stress response by modulating androgen-related pathways other than megalin could decrease KM-induced hearing loss. FM administration reportedly reduced toxic oxidizing radicals, inflammation, and apoptotic cells in a heatstroke mouse study and sepsis models [22,23]. In addition to modulation by megalin expression, androgen and androgen receptors could impact KM-induced ototoxicity via interactions with other drug transporters [24,25]. Reportedly, androgen regulates the expression level of organic cation transporter 2 in rats [24]. Furthermore, FM upregulates estrogen receptors, which may increase estrogen effects [26]. In a rat study using a hemorrhagic trauma model, increased estrogen receptor expression, with no androgen receptor expression, was observed following FM treatment [26]. Although the auditory brainstem response (ABR) thresholds were measured, the otoacoustic emission results were lacking in the present study. Further study with comprehensive auditory measures and diverse dose schedules of FM will warrant clinical therapy in hearing loss patients.

## 4. Materials and Methods

**Figure 6:**
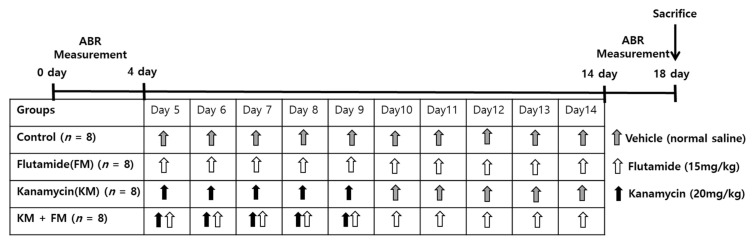
The dosing schedule of the experiments. Rats were divided into four groups: control, kanamycin (KM), flutamide (FM), and KM + FM groups. Auditory brainstem responses (ABRs) were measured before and after completion of drug treatments.

The Institutional Animal Care and Use Committee of CHA University (IACUC200025: accepted date, 6 December 2019) approved the performed animal experiments. The conditions of animal rearing, drug administration, and sacrifice complied with the regulations of the Institutional Animal Care and Use Committee of CHA University. In total, 32 male, 8-week-old Sprague-Dawley rats were used in the present study (Figure 6). The rats were divided into four groups: control, kanamycin (KM), FM, and KM + FM. KM (20 mg/kg/day) was intraperitoneally injected for 5 days in the KM group. In the FM group, 15 mg/kg/day of FM was intraperitoneally injected for 10 days. In the FM + KM group, 20 mg/kg/day of KM and 15 mg/kg/day of FM were intraperitoneally administered for 5 and 10 days, respectively. In the control group, 50 mL/kg of vehicle (normal saline) was intraperitoneally injected for 10 days. Auditory brainstem response (ABR) thresholds were measured before (day 0) and one day after the completion of all drug treatments (days 14–18). The cochleae were harvested one day after ABR measurements (day 18).

### 4.1. Auditory Function Tests

**Figure 7:**
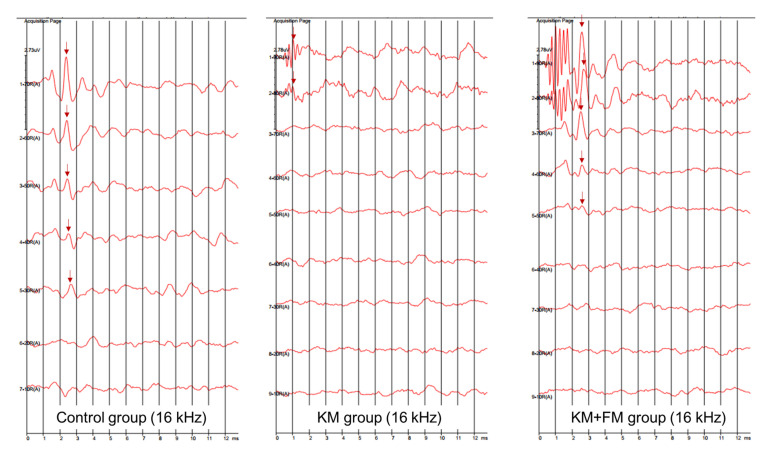
The auditory brainstem response (ABR) traces of control, kanamycin (KM), and KM + flutamide (FM) groups at 16 kHz (arrow: wave II).

The SmartEP system (Intelligent Hearing Systems Corp., Miami, FL, USA) was used to measure ABR thresholds of both ears at 4, 8, 16, and 32 kHz [27]. Anesthesia was induced by intraperitoneally administering a mixture of 40 mg/kg zoletil and 10 mg/kg xylazine. The electrodes were applied to the vertex (reference electrode), contralateral thigh (ground electrode), and ipsilateral retroauricular area (measuring electrode). An EC1 electrostatic speaker was applied to the ipsilateral external auditory canal. Sound stimulation was applied with tone bursts (duration: 1562 µs, Blackman, stimulation rate: 21.2/s, amplitude: 90–20 dB SPL). The auditory brainstem-evoked responses were averaged for 1024 sweeps. The ABR threshold was defined as the lowest sound amplitude detected in wave II [28] (Figure 7).

### 4.2. Cochlear Histologic Examinations

A cochlear whole-mount examination was performed to examine the histology of the outer hair cells. Cochlear whole mounts were prepared from 16 cochleae obtained from eight rats (two rats per group). Harvested cochleae were dipped in 4% paraformaldehyde. Decalcification of the bony labyrinth was performed for 5 days using 120 mM ethylenediaminetetraacetic acid (EDTA). Then, the membranous labyrinth was dissected, and cochlear outer hair cell portions were identified. Following treatment with triton blocking solution, the tissue was stained with 4′ 6-diamidino-2-phenylindole dihydrochloride for 1 h. Then, the tissue was mounted on slides and inspected using a confocal microscope (Zeiss LSM 880, Zeiss, Oberkochen, Land Baden-Wurttemberg, Germany).

H&E staining of the cochlea was performed to evaluate the histology of the organ of Corti and spiral ganglion cells [29]. A total of 16 cochleae from 8 rats (2 rats per group) were examined by H&E staining. In brief, cochleae were fixed in 4% paraformaldehyde solution. After decalcification of the bony labyrinth, the cochlear samples were implanted in a paraffin block. Then, 10 µm-thick sections were obtained and mounted on slides. After deparaffinization and serial washing in ethanol and phosphate-buffered saline, the sections were stained with hematoxylin for 5 min and eosin for 45 s. The stained tissue slides were examined using an EVOS^TM^XL Core Imaging System (Invitrogen, Carlsbad, CA, USA, #AMEX1000). The percentage of intact outer hair cells was counted in the middle turns of cochleae [30].

### 4.3. mRNA Expression Levels of Megalin, Cytochrome P450 1a1 (cyp1a1), and cyp1b1

A total of 32 cochlear tissues from 16 rats (4 rats per group) were used for quantitative reverse transcriptase-polymerase chain reaction (qRT-PCR). The harvested cochlear tissues were stored in a NO_2_ deep freezer. A day after cochlear harvest, total RNA was purified using TRIzol^TM^ Reagent (Invitrogen, Waltham, MA, USA). The quality of extracted RNA was examined using a micro-UV-Vis spectrophotometer (Lifereal Biotechnology Corp. Ltd., Hangzhou, China). The tissues that satisfied the 260/280 ratio (>1.8) and 260/230 ratio (>1.5) were used for experiments. Reverse transcription was performed using Maxime^TM^ RT Pre Mix (Oligo(dT)15 Primer; iNtRON Biotechnology, Seongnam, Korea). The ViiA7 RT-PCR system (Applied Biosystems, Carlsbad, CA, USA) was run using TOPrealTM qPCR 2× PreMIX (SYBR Green with low ROX; Enzynomics, Daejeon, Korea). The forward and reverse primers for megalin, cyp1a1, and cyp1b1 are described in Table 2. HotStarTaq^®^DNA polymerase was activated at 95 °C for 15 s and then at 72 °C for 15 s. For each amplicon, the amplification efficiency was assessed using diluted complementary DNA, as previously described [27]. The mRNA levels were normalized based on the mRNA level of glyceraldehyde 3-phosphate dehydrogenase using the 2^−∆∆Ct^ formula. Fold changes in mRNA levels of each group compared to those of the control group were estimated.

### 4.4. Protein Expression Levels of Megalin, MT1A, MT2A, TNFα, Caspase 3, and Cleaved Caspase 3

In total, 48 cochlear tissues from 24 rats (6 rats per group) were used for Western blotting. Cochlear tissues were lysed in radioimmunoprecipitation assay buffer (Cell Signaling Technology, Danvers, MA, USA). A Bio-Rad Protein Assay Kit was used to measure the protein concentration. Purified proteins were subjected to 8% sodium dodecyl sulfate-polyacrylamide gel electrophoresis. The gels were transferred to polyvinylidene difluoride membranes (Merck Millipore, Burlington, MA, USA) and dipped in blocking buffer (5% nonfat dry milk in Tris-buffered saline containing Tween-20) for 1 h. The membranes were incubated with 1:1000 of anti-megalin (Santa Cruz, Dallas, TX, USA, Sc515772), anti-MT1A (Novus, NBP1-97493), anti-MT2A (MyBioSource, San Diego, CA, USA., MBS8291903), anti-TNFα (Abcam, Cambridge, UK, Ab6671), anti-caspase 3 (Cell Signaling, Danvers, Miami, FL, USA, 9662s), anti-cleaved caspase3 (Cell Signaling, Danvers, Miami, FL, USA, 9661s), and anti-rabbit monoclonal β-actin (Cell Signaling Technology, Danvers, MA, USA, D6A8). Then, membranes were incubated in horseradish peroxidase (HRP)-conjugated secondary antibodies (anti-rabbit IgG, HRP-linked; Cell Signaling Technology, #7074S and goat anti-mouse IgG H&L (HRP); Abcam, Cambridge, UK, #ab97023). Protein bands were evaluated using an enhanced chemiluminescence kit (Bio-Rad, Hercules, CA, USA). Protein bands were quantified using ImageJ software (National Institutes of Health, Bethesda, MD, USA). Protein expression levels of each gene were normalized to those of β-actin. For each group, fold changes in protein expression levels were calculated and compared with those of the control group.

### 4.5. Statistical Methods

The ABR thresholds and mRNA and protein expression levels of control, FM, KM, and KM + FM groups were compared using ANOVA with Bonferroni corrections. The paired *t*-test was used to compare pre- and post-treatment ABR thresholds for each group. The unpaired *t*-test was used to compare ABR thresholds and mRNA and protein expression levels between groups. Statistical significance was set at *p* ≤ 0.05. Data analysis was performed using SPSS version 21.0 (IBM Corp., Armonk, NY, USA). The graphs are presented as mean and error bars (±SD).

## 5. Conclusions

The inhibition of androgen using FM afforded otoprotective effects against KM-induced hearing loss in rats. The otoprotective effects of FM were associated with a decrease in megalin levels. The otoprotective effects of antiandrogen could be one of the reasons for the sex-different responses on aminoglycoside-induced ototoxicity. In further studies with dose adaptation for clinical use, FM could be used to protect or treat the patients with aminoglycoside-induced hearing loss. In addition, because ototoxicity and inflammation are shared pathophysiology of other types of hearing loss, such as noise-induced and other ototoxic drug (e.g., cisplatin)-induced hearing loss, FM can be applied to the treatment of these types of hearing loss.

## Figures and Tables

**Table 1:** The auditory brainstem response (ABR) thresholds at 4, 8, 16, and 32 kHz.

Frequencies		Pre-Treatment			Post-Treatment		
		Mean	Standard error	*p*-value	Mean	Standard error	*p*-value
4 kHz				0.883			0.003 *
	Control	37.50	2.50		38.33	1.67	
	FM	35.00	3.78		37.50	1.64	
	KM	35.00	1.89		53.75	6.80	0.042 † (0.019 **)
	KM + FM	36.25	1.55		36.67	1.81	0.002 ‡
8 kHz				0.659			0.014 *
	Control	37.50	4.53		43.33	2.11	
	FM	36.25	5.96		41.25	5.15	
	KM	42.50	1.64		56.25	4.98	0.033 † (0.020 **)
	KM + FM	39.38	1.70		42.14	1.14	0.021 ‡
16 kHz				0.320			0.005 *
	Control	31.25	2.95		33.33	2.11	
	FM	41.25	2.95		36.25	3.75	
	KM	30.00	3.78		47.50	5.90	0.048 † (0.026 **)
	KM + FM	30.63	1.93		30.00	1.48	0.003 ‡
32 kHz				0.741			0.014 *
	Control	51.25	5.49		61.67	3.07	
	FM	51.25	3.98		57.50	4.53	
	KM	53.75	2.63		75.00	5.00	0.049 † (0.002 **)
	KM + FM	56.25	3.40		59.29	2.67	0.023 ‡

FM: flutamide, KM: kanamycin, * *p* < 0.05 in ANOVA analysis among control FM, KM, and KM + FM groups, ** *p* < 0.05 in paired *t*-test between pre- and post-treatment ABR thresholds. † *p* < 0.05 in Bonferroni correction between control and KM groups. ‡ *p* < 0.05 in Bonferroni correction between KM and KM + FM groups.

**Table 2:** Oligonucleotide primer sequences for quantitative reverse transcriptase polymerase chain reaction.

Gene	Primer Sequence (Forward)	Primer Sequence (Reverse)	Annealing Temperature (°C)	Product Size (bp)	Sequence Number
*Megalin*	5′- TAGCGATTTGGTTCTCCACC -3′	5′- ACTTGTTGGCCTGCATAACC -3′	60	101	NM_030827.2
*Cyp1a1*	5′-ATGTCCAGCTCTCAGATGATAAGGTC-3′	5′-ATCCCTGCCAATCACTGTGTCTAAC-3′	60	167	NM_012540.3
*Cyp1b1*	5′-AAGTCTTGCAGAGTCTGGGC-3′	5′-TGGCCATGCTGCGATGAA-3′	60	92	NM_012940.2
*Gapdh*	5′-AGTGCCAGCCTCGTCTCATA-3′	5′-AAGAGAAGGCAGCCCTGGTA-3′	60	93	NM_017008.4

## Data Availability

The data presented in this study are available upon request from the corresponding author.
